# Editorial: Artificial intelligence-of-things (AIoT) in precision agriculture

**DOI:** 10.3389/fpls.2024.1369791

**Published:** 2024-01-26

**Authors:** Yaqoob Majeed, Longsheng Fu, Long He

**Affiliations:** ^1^ Department of Food Engineering, University of Agriculture Faisalabad, Faisalabad, Punjab, Pakistan; ^2^ Texas A&M AgriLife Research, Texas A&M University System, Dallas, TX, United States; ^3^ College of Mechanical and Electronic Engineering, Northwest A&F University, Yangling, Shaanxi, China; ^4^ Department of Agricultural and Biological Engineering, Pennsylvania State University, University Park, PA, United States

**Keywords:** precision agriculture, internet-of-things (IoT), artificial intelligence, deep learning, digital agriculture, machine vision

Precision agriculture is becoming critically important for sustainable food production to meet the growing food demand. In recent decades, technical advances in AI (artificial intelligence) and IoT (internet-of-things) can help solve various agricultural field problems and optimize resource utilization (e.g. water, pesticide, fertilizer, seed, energy), improve production management and productivity, and reduce labor dependency. AI and IoT-enabled applications are increasingly implemented for precision agriculture applications such as crop growth monitoring, weed removal control, pest and disease detection, planting, crop yield estimation, targeted spraying and pollination, smart irrigation and nutrient management, field analysis, and plant phenotyping. For example, IoT-based applications using machine learning and deep learning models are widely used to recognize fruits, vegetables, weeds, pests, and diseases, and measure soil quality and nutrients. Such information helps inform better crop management practices. Despite the progress of AI and IoT technologies in precision agriculture, the combined use of these technologies in the form of AIoT are still in early stages with numerous challenges in the form of data acquisition and connectivity, and optimization of AI algorithms based on edge computing processing capabilities that still need to be addressed.

This Research Topic focuses on the recent advancement in the area of AI and IoT applications on precision agricultural technologies for both field and specialty crops. This Research Topic attracted nine research articles and three review articles. These articles reveal the research advancements and trends of applied machine learning and deep learning techniques for various precision agriculture applications.

Robotic harvesting plays an important role in addressing the labor shortage problems for manual labor-intensive and time-sensitive harvesting operations. For example, Sun et al. propose the YOLO-P to detect the pears for robotic harvesting in natural orchard environment. They propose the shuffle block integrated with convolutional block attention module (CBAM) as the backbone of YOLOv5 network. A total of 5,257 images consisting of various backgrounds and illumination conditions were used to train and test the proposed approach. Different ablation experiments were performed to check the robustness and generalization and obtained the 0.961 F1-score with 32 FPS (frames per second). To facilitate autonomous driving of robot and roadside fruit harvesting, Zhou et al. proposed the framework for synchronous road extraction and roadside fruit recognition. Gray factor optimization approach was adopted to extract the unstructured roads from images while YOLOv7 was employed to detect the wine grapes. The proposed synchronous approach helped to increase fruit detection by 23.84%.

In another study, Tang et al. estimated the tree-level almond yield using aerially captured multispectral images and convolutional neural networks. They used approximately 2000 almond trees for the yield monitoring. Multispectral aerial images were collected at a height of 6,000 ft with 0.3 m spatial resolution. Then, convolutional neural network (CNN) with spatial attention module was proposed to estimate the yield estimation at tree-level. Their proposed approach achieved the R^2^ and RMSE (root mean square value) of 0.96 and 6.6%, respectively. Similarly, Ren et al. introduce the mobile robotic platform for indoor farming to monitor strawberry yield. They first developed the autonomous mobile robot platform (AMR) that uses the AprilTag and inertial navigation to autonomously navigate the structural environment of indoor farms. Then, they used the multilayer perception robot (MPR), mounted on ARM, to collect the temporal-spatial data of the strawberry plants within the strawberry indoor farm. Their MPR achieved the positioning accuracy of 13.0 mm while navigating the plant factory with 6.26% error rate in yield monitoring performance.

Precision pest management is another area in precision agriculture which involves accurate pest detection and identification for the precise pesticide applications. For example, Peng et al. employed an ensemble learning technique to fuse the selective kernel unit, representative batch normalization module, and ACON activation with the Dense-Net-121 networks, naming it MADN, to detect and identify the crop pests. Their proposed approach helped to achieve F1-score of 0.7528 in identifying the pests.

To optimize coconut breeding, Liu et al. introduced a non-destructive approach to segment the internal organs of coconuts using Computed Tomography (CT) scanning and semantic segmentation. They scanned the coconut during different stages using the CT scan and constructed the CIDCO dataset. Then DeepLabv3+ based semantic segmentation was employed by introducing dense atrous spatial pyramid pooling and CBAM modules. Their improved model helped to achieve F1-score of 0.905 to segment the internal organs of coconuts. Similarly, non-destructive and automatic detection of defective kiwifruit is critically important to maintain the postharvest quality of kiwifruits and for consumer acceptability. To address this issue, Wang et al. focused on detecting the defective kiwifruits for grading lines by employing YOLOv5. They constructed a multiple-defect kiwifruit dataset consisting of healthy, leaf-rubbing, damaged, healed cuts or scarred, and sun-burn kiwifruits. Then, spatial-depth and depth-wise separable convolutional modules were combined with YOLOv5 to improve the detection performance of the defective kiwifruits. Their approach helped to achieve an average detection accuracy of 97.7% with 8.0 ms detection time.

It has been always a challenge for dataset availability and its manual labeling to train AI based algorithms to solve the specific precision agriculture application. To address this problem, Wang et al. introduce a deep reinforcement learning based augmentation framework for the leaf rust images. Their proposed approach consists of Deep Q-Learning (DQN) for selecting optimal augmentation approach based on individual image, extracting geometric and pixel indicators, and DeepLabv3+ to authenticate augmented image and feedback the rewards. Experimental results showed that the proposed approach helped to achieve Intersection-over-Union (IoU) of 0.8426 in correctly classifying leaf rust spots compared to the union of expected and predicted rust spots.

Measurement of plant phenotypic traits is critical in selecting the high-yield crop varieties and timely identifying the need in actions for optimal plant growth. To measure the soybean plant phenotyping traits, He et al. proposed a generalized regression neural network based approach. First, SfM (structure from motion) algorithm was used to reconstruct the soybean plants. Then, different filtering (lowpass filter and gaussian filter) and Laplacian smoothing methods were used to segment different parts of soybeans (e.g. plants, stem, and leaves). Ultimately, a generalized regression neural network was employed to measure the phenotypic traits of the soybeans. Results indicated that their proposed approach helped to achieve R^2^ of 0.9775, 0.9785, and 0.9487 for measuring the plant height, lead length, and leaf width, respectively compared to ground truth measurements.

In addition to the above-mentioned studies, there are further areas in which AI-assisted technologies could be used for precision agriculture applications. For example, Nawaz et al. reviewed the latest trends in applying data processing and deep learning algorithms for remote sensing data. Furthermore, Estrada et al. explored and reviewed machine learning applications for remote forestry health assessment. Similarly, Johnson & Cheein presented a comprehensive review on the use of mechatronics, AI and IoT applications for potato harvesting.

With the papers published in this Research Topic ranging from different precision agriculture applications and covering latest advancements in the AI application to solve various agricultural challenges, we hope readers will gain insights into the state-of-the-art developments in rapidly growing precision and digital agriculture domain and will provide further opportunities for scientists and industries to take on the collective challenges faced by this sector. The papers published in this Research Topic proved the critical role of AI and IoT applications to address global food security issues and meet the sustainable agriculture goals in the context of declining and aging agricultural labor. However, more studies will be needed with continuous innovations, and collective efforts from scientists and industries working in the precision and digital agriculture domain.

## Author contributions

YM: Writing – original draft, Writing – review & editing. LF: Writing – review & editing. LH: Writing – review & editing.

